# Supplementary material for: Tests of the Oddity Effect Hypothesis in mixed-species parid flocks
Source: PLoS One. 2026 Jan 30;21(1):e0341779. doi: 10.1371/journal.pone.0341779 (PMC12858019; doi:10.1371/journal.pone.0341779)
Supplement: Attachment — Submitted filename: Response to Reviewers.docx [file pone.0341779.s003.docx]

Response to Reviewers – Plos ONE (PONE-D-25-21798)

Tests of the Oddity Effect Hypothesis in mixed-species parid flocks

Dear Dr. Mazza:

Thank you for giving us the opportunity to revise and resubmit our manuscript to

address the helpful criticisms and questions of the reviewers. We have copied and

pasted the entire set of comments of the reviewers, which we include below. For each

comment, we briefly describe in red text how it was addressed and, if relevant, where

the changes can be found (by line number) in the clean, non-track-changes manuscript.

+++++++++++++++++++++++++

Thank you for submitting your manuscript “Tests of the Oddity Effect Hypothesis in mixed-species parid flocks” to PLOS One. I regret that I am unable to accept your paper for publication in Plos One in its present form. However, I would be prepared to consider for publication a substantially revised version that takes into account the suggestions of the reviewers. Such a revised paper would be reviewed and there would be no guarantee of acceptance.

We thank you and the reviewers for the opportunity to resubmit our manuscript, regardless of the end decision!

When submitting your revision, we need you to address these additional requirements.

1. Please ensure that your manuscript meets PLOS ONE's style requirements, including those for file naming. The PLOS ONE style templates can be found at

https://journals.plos.org/plosone/s/file?id=wjVg/PLOSOne_formatting_sample_main_body.pdf  and

https://journals.plos.org/plosone/s/file?id=ba62/PLOSOne_formatting_sample_title_authors_affiliations.pdf

2. Please include captions for your Supporting Information files at the end of your manuscript, and update any in-text citations to match accordingly. Please see our Supporting Information guidelines for more information: http://journals.plos.org/plosone/s/supporting-information.

Thank you for these comments, they have been addressed in the updated manuscript!

**Reviewer 1:**

**Is the manuscript technically sound, and do the data support the conclusions?**

The manuscript must describe a technically sound piece of scientific research with data that supports the conclusions. Experiments must have been conducted rigorously, with appropriate controls, replication, and sample sizes. The conclusions must be drawn appropriately based on the data presented.

Reviewer #1: Partly

We thank the reviewer for the comment and hope we have expanded our manuscript to fully meet the requirements for this specification.

**Has the statistical analysis been performed appropriately and rigorously?**

Reviewer #1: No

Thank you for this comment, it has been addressed in the updated manuscript in Lines 194-212. We opted to keep the non-parametric approach given its simplicity and conservative nature for studies with small Ns. We note below and in the text, however, that GLM analyses resulted in similar outcomes.

**Have the authors made all data underlying the findings in their manuscript fully available?**

The PLOS Data policy requires authors to make all data underlying the findings described in their manuscript fully available without restriction, with rare exception (please refer to the Data Availability Statement in the manuscript PDF file). The data should be provided as part of the manuscript or its supporting information, or deposited to a public repository. For example, in addition to summary statistics, the data points behind means, medians and variance measures should be available. If there are restrictions on publicly sharing data—e.g. participant privacy or use of data from a third party—those must be specified.

Reviewer #1: Yes

We thank the reviewer for the supportive comments!

**Is the manuscript presented in an intelligible fashion and written in standard English?**

PLOS ONE does not copyedit accepted manuscripts, so the language in submitted articles must be clear, correct, and unambiguous. Any typographical or grammatical errors should be corrected at revision, so please note any specific errors here.

Reviewer #1: Yes

We thank the reviewer for the supportive comments!

**Please use the space provided to explain your answers to the questions above. You may also include additional comments for the author, including concerns about dual publication, research ethics, or publication ethics. (Please upload your review as an attachment if it exceeds 20,000 characters)**

***General comments***

This is a manuscript regarding a very interesting topic, not very investigated. However, I found some weaknesses about clarity of methods and results. In particular, you tested the oddity effect hypothesis with very small sizes (i.e. N = 2) for sole birds and although you used non parametric tests, you should be very cautious to draw conclusions.

We thank the reviewer for the supportive comments and hope we have addressed all concerns adequately below!

***Specific comments***

*Methods*

1. Line 150-157 and 178-182: if I understand correctly, in both experiments, you had to wait 10-15 minutes before showing the predator model mount, in which you count birds in the flock. Then you walked to the feeder, placed the model, and returned to the observation place. Don’t you think that the operator could have affected in some way the birds behavior? Do birds remained there, at the feeder, or flew away when you walked to place the predator models? I think that it is important to write something about it, either if operator was disturbing or not.

We agree and have added a few sentences to reflect this in Lines 142-152, 160-163, and 170-182. We conducted the same movements during the pre-stimulus and stimulus periods to avoid affecting the birds’ behaviors as much as possible. For the pre-stimulus period, we walked to the feeding platform (screech owl experiment) or model mount (Cooper’s hawk experiment) and touched it, and for the stimulus period, we walked to the feeding platform/model mount and placed the model on top. Also, the birds in our observation areas are used to observers so although they usually avoid the feeding platform when observers get too close (during both the pre-stimulus and stimulus periods) they rarely leave the area. The birds are typically back to foraging at normal rates from the feeders within seconds of our leaving the immediate area of the feeders.

2. Line 157: how far was the observation place in the screech owl experiment? Was the observer hidden or in plain sight? Please, add these details.

Thank you for catching this, we have addressed the observation distance and visibility in Lines 142-146 and 161-163.

*Results*

3. Lines 205-208: so, to test both the “probability of calling” and the “recruitment” hypotheses, you correlated the same things, i.e. number of conspecifics in the flock vs the latency for the first individual to call. What differed was the prediction, right? Aren’t these hypotheses in contrast? When a bird is rare in a mixed flock either could be silent to doesn’t draw attention (probability of calling hypothesis) or be loud to recruit conspecifics (recruitment hypothesis). I suggest to say something about this, also in discussion, if there is some threshold that lead to one or the other hypothesis, and why a rare bird choose to be quite or loud.

Thank you for this suggestion! Yes, these hypotheses have contrasting predictions. The recruitment hypothesis is more about call rate than volume, but we have added text to the Introduction (Lines 101-113), Methods (Lines 208-212) and Discussion (Lines 362-378) sections to address these comments.

4. Lines 223-225 and 235-242: as I understand, you compared latencies of 2 vs 34 chickadees flocks in the screech owl experiment, latencies of 2 vs 28 chickadees flocks in the hawk experiment, and latencies of 2 vs 28 titmice flocks in the hawk experiment. Therefore, even if you used non-parametric tests, I suggest to be very cautious to draw some conclusions with these results for the oddity effect hypothesis.

We have adjusted the language to reflect consideration of our small sample size (Lines 275-276, 286, 349-352, and 379-381).

*Discussion and Conclusions*

5. As I said in the previous comment, because of the small sample size you should be very careful to draw conclusions about the oddity effect hypothesis, as you said as well in conclusions. However, you seemed very convinced of these results (see lines 263-265, 277-279, 282, 299), but I suggest to rephrase using a more cautious tone.

We have adjusted the language to reflect consideration of our small sample size (Lines 275-276, 286, 349-352, and 379-381), as mentioned earlier.

**Reviewer #2:**

**Is the manuscript technically sound, and do the data support the conclusions?**

Reviewer #2: Partly

We thank the reviewer for the comment and hope we have expanded our manuscript to fully meet the requirements for this specification.

**Has the statistical analysis been performed appropriately and rigorously?**

Reviewer #2: No

Thank you for this comment, it has been addressed in the updated manuscript in Lines 194-212.

**Have the authors made all data underlying the findings in their manuscript fully available?**

Reviewer #2: Yes

We thank the reviewer for the supportive comments!

Is the manuscript presented in an intelligible fashion and written in standard English?

Reviewer #2: No

We are not sure what the reviewer means by this comment, but would be happy to address any unclear or confusing text in our manuscript.

**Review Comments to the Author**

**General comments:** I think this is a nice study, and a good addition to the growing body of literature that is trying to dig into individual behavioral changes in flocking contexts. I do, however, think that the abstract, introduction, and methods could all use some restructuring to make both the question, statistics and field methods clearer. In particular, it is hard to understand exactly what data were taken, whether the data between the two experiments is different, and what the purpose of the two experiments was. I also would strongly consider the authors changing their statistical analysis to take advantage of GLMs and the fact that they can incorporate multiple factors and interactions. I think this reframing in a way that unifies the data and the variability present in the mixed-species flocks may significantly impact the discussion as well.

We thank the reviewer for the supportive comments on our study and hope that we have adjusted our manuscript to address these concerns!

***Abstract:***

Line 15 - This first sentence is confusing and, as it stands, could be interpreted multiple ways. I would advise reframing this and maybe swapping the major points of the first two sentences in the abstract. First, describe rarity, then describe its role in predation.

We have changed the wording to reflect this in Lines 16-19.

Line 25 - The description of the experiment here is also a bit confusing. Given this is the abstract, there isn't much space to explain further, but the details added here don't clarify your findings and instead pose several questions. Why two experiments? How did they differ? Were they analyzed together or separately? Given that (after going through the rest of the paper) the data for the two experiments are different yet are addressing the same question in very similar ways I would just simplify this and state that you had evidence that at least two species (chickadees and titmice) were less likely to call (alarm call?) in response to predators when alone as compared to with conspecifics. I would then maybe use that tiny bit of extra space to explain further the comment below.

Thank you for the suggestions, we have added clarification in Line 22-23.

Line 27-28 - I also had trouble following this explanation of ruling out alternative hypotheses. Are these your alternatives, or is this what you found? Maybe stating that you tested both the oddity effect and these two alternative hypotheses in one sentence earlier in the abstract would help clarify.

Thank you for the suggestions, we have added clarification in Lines 26-27 and 30-33.

***Introduction:***

Line 65 - Somewhere here I think it is important to state that mixed-species groups are often signaling to each other - transmitting information about food or predators etc. This will set up the reasoning below that it isn't always good for specific (rare) individuals to call.

Thank you for this – we have added some clarification to Lines 50-52.

Line 67 - might be better here to emphasize that signaling can increase predation by making individuals more conspicuous, prior to describing how a reduction in signaling can mitigate this.

Yes, we have added some clarification to Lines 52-56.

Line 75 - 76 - sentence here about attracting mates and deterring competitors feels out of place. somewhere here, it might be better to lean into the mixed-species group stuff and talk about how there may be benefits to signaling in flocks, but that rarity may interact with appearance/vocal distinctiveness to make it not always beneficial and thus a contributing factor to flock formation, function, and individual behavior

We have included this suggestion to Lines 66-67.

Line 86 - mixed species should have a dash in it

Yes, thank you for catching this! This has been added to Line 71.

Line 105 - single conspecifics within heterospecific flocks? And multiple conspecifics within heterospecific flocks? Assuming yes, but this should be clear.

We have added some clarification to Lines 90-93.

Line 117 - 121 - describing the hypothetical probabilities here seems unnecessary. I would in stead use this space to describe your second alternative hypothesis, perhaps describing the recruitment of birds to mobbing flocks more explicitly. May also expect that birds would call to recruit in order to avoid the oddity effect.

We have included these suggestions in Lines 105-113.

***Methods:***

General comment - I'd recommend putting a sentence in near the front of the methods here clearly stating you conducted two experiments using two different predator stimuli. The first you mention that there were two is "Our two experiments involved..."

We have added some clarification to Line 118.

Also would be helpful to clarify why you used these two different experiments. Is it because these two predators represent different threat levels?

Yes, this has been added to Lines 130-131.

Line 146 - It is implied, but not clear, that you also used this same plastic screech owl that was used in the other study. Also might be good to state that in the previous work birds responded to it like a predator, as many birds get habituated to plastic stimuli.

We mention the reactivity of our study species to the screech owl model in Lines 140-141.

Line 152 - are you assuming all the individuals remained in the area following the placement of the stimuli? Did you account for any individual departures driven by the presence of the observer with the stimulus?

As addressed from Reviewer 1’s suggestions, clarification has been added to Lines 142-152, 160-163, and 170-182, and 180-183 to address pre-stimulus and stimulus bird behavior.

Line 157 - Was the call type recorded? Also, assuming you recorded what species was producing each call.

Thank you for catching this! The species that called during the 10-minute stimulus period was voice-recorded and this has been clarified in Lines 153-155 and 160-163.

Line 184 - What do you mean by "coded"? Identified? Did you record the time to the first call as well as the number of each call type?

Yes, we added clarification to Line 184-188. As mentioned in Line 194-195, we recorded the time (latency) to call first, not including the number of each call type.

General method question: Were each of the experiments only conducted once at each of the 36 feeder sites?

Yes, each experiment was only conducted once at each of the sites. This has also been added to Line 120.

Line 195 - explain how the data were non-normal

We added some text to address this – Lines 195-199. In short, Kolmogorov-Smirnov one-sample tests all indicated the distributions of the latency to call data were statistically different from a normal distribution and the standard deviations were all larger than the means.

Line 194 - This paragraph could use a bit more structure. For example, consider telling us how many analyses you ran to answer what questions. Even having a list of 1, 2, 3 might help. It is also not clear what was analyzed for which data set. Were they all compiled?

We are not sure what the reviewer means here but tried to revise the text here to make things clearer – Lines 199-212.

General methods question - given that there is likely a variable number of both heterospecifics and conspecifics in each of these experimental flocks, I'd recommend using GLMs or a similar analysis that would allow you to incorporate both the number of conspecifics and heterospecifics and the potential interaction between them into your analysis. You could also include species and put all the species into the same analysis.

This information is partly addressed in the two other studies that were the foundational studies for the analyses here. We have kept the analyses simple in the present manuscript but have added a bit more contextual information. We also note that we analyzed the data sets here using GLM with negative binomial distribution and get the same qualitative results – the significant effects for titmice and nuthatches in the Coopers hawk model study and for chickadees in the screech owl model study remain (Lines 194-199).

Line 212 - The word ethically here isn't informative. Maybe instead say "Experiments were observational and did not involve the handling of any individuals. While predator stimuli can be temporarily stress-inducing, predator encounters are a normal occurrence, and thus these experiments are considered benign."

Thank you for this suggestion, we have revised the sentence in Lines 215-218.

Line 216 - consider rephrasing this: "In the screech owl experiment, flocks consisted of..." Can you also include the mean flock size over all for each experiment?

The rephrasing has been added to Line 221 and the mean flock sizes in Lines 223-226.

Line 249 - you say there was no significance, but report a p value of 0.015 for nuthatches. is this a typo?

The direction of the effect is opposite of the prediction (significant positive correlation between calling latency and the number of conspecifics), and so the hypothesis is not supported.
