# Supplementary material for: Tests of the Oddity Effect Hypothesis in mixed-species parid flocks
Source: PLoS One. 2026 Jan 30;21(1):e0341779. doi: 10.1371/journal.pone.0341779 (PMC12858019; doi:10.1371/journal.pone.0341779)
Supplement: Attachment — Submitted filename: Response_to_Reviewers_auresp_2.docx [file pone.0341779.s004.docx]

Response to Reviewers – Plos ONE (PONE-D-25-21798R1)

Tests of the Oddity Effect Hypothesis in mixed-species parid flocks

Dear Dr. Mazza:

Thank you for giving us the opportunity to revise and resubmit our manuscript again. We have copied and pasted the entire set of comments of the reviewers, which we include below. For each

comment, we briefly describe in red text how it was addressed and, if relevant, where

the changes can be found (by line number) in the clean, non-track-changes manuscript.

+++++++++++++++++++++++++

Thank you for submitting your manuscript to PLOS ONE. After careful consideration, we feel that it has merit but does not fully meet PLOS ONE’s publication criteria as it currently stands. Therefore, we invite you to submit a revised version of the manuscript that addresses the points raised during the review process

We thank you and the reviewers for the opportunity to resubmit our manuscript, regardless of the end decision!

Thank you for your revisions. Both previous reviewers kindly agreed to read the new version, and both agree that the manuscript is substantially improved. One reviewer noted the somewhat inconsistent way in which the oddity effect hypothesis is formulated, and I agree this aspect should be addressed according to the reviewers' recommendations. Both reviewers also noted a couple of minor formal point to straighten up. I am sure these aspects will take very little time to address, and I look forward to your revised submission.

If the reviewer comments include a recommendation to cite specific previously published works, please review and evaluate these publications to determine whether they are relevant and should be cited. There is no requirement to cite these works unless the editor has indicated otherwise. 

Please review your reference list to ensure that it is complete and correct. If you have cited papers that have been retracted, please include the rationale for doing so in the manuscript text, or remove these references and replace them with relevant current references. Any changes to the reference list should be mentioned in the rebuttal letter that accompanies your revised manuscript. If you need to cite a retracted article, indicate the article’s retracted status in the References list and also include a citation and full reference for the retraction notice.

Thank you for these comments, they have been addressed in the updated manuscript! Our references have also remained the same.

**Comments to the Author:**

**If the authors have adequately addressed your comments raised in a previous round of review and you feel that this manuscript is now acceptable for publication, you may indicate that here to bypass the “Comments to the Author” section, enter your conflict of interest statement in the “Confidential to Editor” section, and submit your "Accept" recommendation.**

Reviewer #1: All comments have been addressed

Reviewer #2: (No Response)

We thank the reviewers for their comments and hope we have expanded our manuscript to fully meet their suggestions.

**Is the manuscript technically sound, and do the data support the conclusions?

The manuscript must describe a technically sound piece of scientific research with data that supports the conclusions. Experiments must have been conducted rigorously, with appropriate controls, replication, and sample sizes. The conclusions must be drawn appropriately based on the data presented.**

Reviewer #1: Yes

Reviewer #2: Yes

We thank the reviewers for their comments.

**Has the statistical analysis been performed appropriately and rigorously?**

Reviewer #1: Yes

Reviewer #2: Yes

We thank the reviewers for their comments.

**Have the authors made all data underlying the findings in their manuscript fully available?**

The PLOS Data policy requires authors to make all data underlying the findings described in their manuscript fully available without restriction, with rare exception (please refer to the Data Availability Statement in the manuscript PDF file). The data should be provided as part of the manuscript or its supporting information, or deposited to a public repository. For example, in addition to summary statistics, the data points behind means, medians and variance measures should be available. If there are restrictions on publicly sharing data—e.g. participant privacy or use of data from a third party—those must be specified.

Reviewer #1: Yes

Reviewer #2: Yes

We thank the reviewers for their supportive comments!

**Is the manuscript presented in an intelligible fashion and written in standard English?**

PLOS ONE does not copyedit accepted manuscripts, so the language in submitted articles must be clear, correct, and unambiguous. Any typographical or grammatical errors should be corrected at revision, so please note any specific errors here.

Reviewer #1: Yes

Reviewer #2: Yes

We thank the reviewers for their comments.

**Review Comments to the Author**

*Reviewer #1*:

This version of the manuscript is greatly improved by following reviewers' suggestions. I found only some minor issues that need to be addressed before acceptance:

Line 17: “one” what? Maybe “one individual” or “one bird” or similar is better.
Line 142: “Similar movements” of what/who? Please, replace with something like “Observers moved in the same way” or “We moved in the same way” to be more clear
Line 362: remove the p-value from the conclusion

Nice work!

We thank the reviewer for the supportive comments and have addressed these comments in Lines 17, 145-146, and 372.

*Reviewer #2*:

Thank you for addressing my previous recommendations, I think the manuscript is now much stronger and clearer. I do want to apologize for the comment that said the manuscript wasn't presented in an intelligible fashion, that was me clicking incorrectly in the dropdown menu and not realizing I had hit no when I had meant yes.

I only have three minor wording suggestions that you can take or leave and one bigger comment that I strongly recomend you consider (the first comment below). Otherwise I think the manuscript is sound!

We thank the reviewer for the supportive comments and completely understand the mishap, thank you for your clarification!

Line 53—be careful about defining the oddity effect, it is typically defined by predators being attracted to odd, distinctive prey items within a group, but here you are making it sound like it is about prey behavior. While I don’t fully disagree with the definition, I think it might be better to define the oddity hypothesis in the context of the predator at the start of this paragraph then reframe how you are referring to the prey behavior throughout as a response to oddity or a reduction of risk. For instance, on line 284 you give the example from pigeon predation choice by goshawks and also call it the oddity effect, but this is a very different definition that you present at the start and is more in line with the general literature. I would recommend laying out your terms more clearly and to more appropriately reflect how this phrase is typically used, and make that change in framing throughout the manuscript.

We thank the reviewer for this suggestion and have addressed it in Lines 21-23, 53-57, 99-100, 115-116, 120, 202, 297-298, 314-315.

Line 43—Large here is a bit subjective, I’d recommend saying “Groups can often…”

We agree and have changed the wording in Line 43.

Line 68—Common might be better as “well-known”

We have changed the wording in Line 69.

Line 257—Thank you for clarifying my confusion about your p-value in the last revision, I should have noticed the effect size. I do however think it might be important to point out that while you didn’t find a significant positive relationship, you instead found a significant negative relationship. It will help to emphasize the finding and also jog the reader to recognize what that finding means, like I needed!

We agree and have added this suggestion to Lines 260-263.
